# Secondary metabolites produced by endophytic fungi, *Alternaria alternata*, as potential inhibitors of the human immunodeficiency virus

**DOI:** 10.3389/fgene.2022.1077159

**Published:** 2022-12-13

**Authors:** Bruce Nzimande, Hezekiel M. Kumalo, Sizwe I. Ndlovu, Nompumelelo P. Mkhwanazi

**Affiliations:** ^1^ Discipline of Medical Microbiology, School of Laboratory Medicine and Medical Sciences, Medical School, University of KwaZulu-Natal, Durban, South Africa; ^2^ Drug Research and Innovation Unit, Discipline of Medical Biochemistry, School of Laboratory Medicine and Medical Sciences, Medical School, University of KwaZulu-Natal, Durban, South Africa; ^3^ Department of Biotechnology and Food Technology, Faculty of Science, University of Johannesburg, Johannesburg, South Africa; ^4^ HIV Pathogenesis Programme, Doris Duke Medical Research Institute, School of Laboratory Medicine and Medical Sciences, University of KwaZulu-Natal, Durban, South Africa

**Keywords:** *Alternaria alternata*, anti-HIV-1, crude extracts, endophytic fungi, HIV-1, *Hypoxis* species, *Sclerocarya birrea*

## Abstract

Antiretroviral treatment has significantly reduced human immunodeficiency virus infection and mortality. However, the current treatment regimen is limited by adverse side effects, the emergence of drug resistance, and the inability to eliminate viral reservoirs. Here, fifteen endophytic fungi were isolated from *Sclerocarya birrea* and *Hypoxis* plants. Crude extracts of *Alternaria alternata* (strain ID PO4PR1, PO4PR2, and PO2PL1) of the fifteen isolate’s crude extracts showed anti-HIV-1 activity in TZM-bl cell line at inhibitory concentration (IC_50_) values ranging from 0.017 to 1.170 μg/ml. The three crude extracts also maintained the virus replication inhibition profile on PBMCs and CD4^+^ T cells at concentrations ranging from 0.3 to 50.2 ng/ml. Partial purification using the solid phase extraction and analysis with Gas Chromatography-Mass spectrophotometry showed a diverse profile. The bioactive compounds were identified based on peak area, retention time, similarity index. The major compounds from GC-MS analysis of *A. Alternata* revealed the existence of cyclotrisiloxane octamethyl (22.92%); Propaninitrile (16,67%); Pyrrolol[1,2-a]pyrazine-1,4-dione, hexahydro-3-(2-methyl propyl) (10.42%); Silane, diethylethoxy(2-ethoxyethyloxy) (4.17%); Coumarin, 3,4-dihydro-4,5,7-trimethyl- 4,5,7-Trimethyl-2-chromanone (13.7%) and 1,2-Cyclobutanedicarbonitrile (2.08%) with previously reported biological activities such as antimicrobial, anti-inflammatory and antioxidant properties. Therefore, these bioactive compounds from *A. alternata* fungal endophytes could be repurposed as potential anti-HIV agents. This study showed the potential of endophytic fungi, *Alternaria alternata* from *S. birrea,* and *Hypoxis* species as producers of anti-HIV compounds.

## 1 Introduction

The human immunodeficiency virus (HIV) is a public health issue worldwide, particularly in developing countries (Gayle and Hill, 2001; De cock et al., 2012). In 2021, 38.4 million people were living with HIV, which has claimed 40.1 million lives thus far (UNAIDS, 2022). Recent advances in highly active antiretroviral therapy (HAART) have changed the status of the HIV pandemic to a chronic, manageable disease in many areas of the world, thus prolonging HIV-infected patients’ lifespans (Venkatesh et al., 2010). Despite the significant progress in improved access to ARVs, there are still challenges which include the development of drug resistance, serious adverse effects, and the inability to eradicate the virus from viral reservoirs (Geskus et al., 2007; Cihlar and Fordyce, 2016). Therefore, there is an urgent need for new HIV therapies that can escape the existing resistance mechanisms by targeting additional viral proteins that are not targeted by the currently available treatment regimen.

Medicinal plants have been used to manage different diseases or symptoms of viral, bacterial, and fungal infections (Ingale and Hivrale, 2010). Specifically, *S. birrea* is well known for its antibacterial, antihelmintic, antifungal, and anticonvulsant effects. *Hypoxis* species has been documented to treat urinary infections and cardiac diseases. It increases immune function, prostate hypertrophy, burns, wounds, and ulcers (Ncube et al., 2013). In another study conducted by Mills et al. (2005), there is some indirect evidence that the isolated compounds, sterols, and sterolins, from the roots of the *Hypoxis* plant have the potential to enhance immunity (Mills et al., 2005). Recently, the focus has shifted from medicinal plants to their microbiome as a promising source of active secondary metabolites. This makes the bioprospecting of the endophytic fungi inhabiting medicinal plants for secondary metabolites an interesting potential niche for discovering anti-HIV agents. Plant-associated microorganisms, particularly the fungal species, termed endophytic fungi, have gained more interest in modern drug screening platforms, mainly because of the chemical complexity and diversity of natural products which cannot be replicated through synthetic means (Strobel and Daisy, 2003; Nath and Joshi, 2021). Endophytic fungi are microorganisms that colonize the inner tissues of healthy plants without causing any harm to the host plant (Petrini, 1991; Dutta et al., 2014). Endophytic fungi have co-evolved with their host plants throughout endosymbiosis, which has allowed the endophytes and host plants to share genetic materials (Lim et al., 2012; Carroll, 2017). The potential of endophytic fungi to produce similar secondary metabolites to their host plants makes fungal species valuable candidates for the future development of new drugs (Kurapati et al., 2016).

The biologically active secondary metabolites produced by fungi can be exploited for various activities such as anti-inflammatory, antimicrobial, anticancer, and antiviral, including HIV-1 (Roy, 2017; Hyde et al., 2019). Since the discovery of taxol (paclitaxel) from the endophytic fungus *Taxomyces andreanae* in 1993, many scientists have shown a keen interest in studying fungal endophytes as potential producers of biologically active compounds (Stierle et al., 1993). Notably, anti-HIV compounds such as calanolide A, isolated from *Calophyllum lanigerum* of tropical trees distributed in the Indo-Pacific region, have shown significant inhibition of HIV-1 replication (Kashman et al., 1992). Other compounds, such as heteroclitin D, isolated from *Kadsura heteroclita*, have shown moderate anti-HIV activity in C8166 cells (Pu et al., 2008). Several fungal secondary metabolites inhibiting various stages of the HIV-1 life cycle have been identified from various fungal species, including xanthoviridicatins E and F from *Penicillium chrysogenum*, altertoxins from *Alternaria tenuissima*, quinic acid from *Helichrysum mimetwes* and many others (Singh et al., 2003; Bashyal et al., 2014; Yazdi et al., 2019). These findings stimulated interest in exploiting secondary metabolites from endophytic fungi associated with plants to find anti-HIV therapeutics. This study presents the isolation and identification of bioactive compounds isolated from the endophytes, *A. alternata* inhabiting *S. birrea* and *Hypoxis* plant species. In addition, the HIV-1 inhibition activities of the selected crude extracts from these isolates were investigated. Bioactive crude extracts were partially purified followed by chemical profiling using gas chromatography-mass spectrometry (GC-MS).

## 2 Materials and methods

### 2.1 Plant collection

S*. birrea* (voucher no. 18234) and *Hypoxis* species (voucher no. 18233) were collected from the South Coast region of the Durban Municipality, KwaZulu-Natal. The voucher specimens were deposited at the UKZN School of Life Science Herbarium for taxonomic identification.

### 2.2 Isolation of endophytic fungi

A total of fifteen endophytic fungi, four from the roots of *Hypoxis* species, eleven from *S. birrea* (one from the root, four from the leaves and six from the stem) were isolated following a protocol described by Arivudainambi et al. (2011). Briefly, the medicinal plants’ roots, leaves, and stems were washed twice with sterile distilled water. The surfaces were then sterilized in 70% (v/v) ethanol for 1 min and consecutively in 1% (v/v) sodium hypochlorite for 3 min before immersing them in 70% (v/v) ethanol for another minute. The final wash was done in sterile distilled water three times to remove excess sterilant. The plant segments were air-dried under sterile conditions under a laminar flow. After drying, each plant segment was cut into small pieces (5–7 mm) and aseptically plated on Potato Dextrose Agar (PDA) and Malt Extract Agar (MEA) (Neogen, United States) supplemented with 100 μg/ml ampicillin (Merck, South Africa) to inhibit the growth of bacteria. The plates were incubated at 25°C in the dark for 5 days to promote fungal growth. Subsequent purification steps were followed until pure cultures were obtained.

### 2.3 Molecular identification of endophytic fungi

Three of the 15 endophytic fungal isolates (PO4PR1, PO4PR2, and PO2PL1) were selected for molecular identification based on their anti-HIV activity profiles during pre-screening (data not shown). Genomic DNA was isolated using the Norgen Plant/Fungi DNA isolation kit (25240, Norgen Biotek, Thorold, ON, Canada) per the manufacturer’s instructions. The extracted fungal DNA was used for the amplification of the internal transcribed spacer (ITS) sequence region using primers: forward primer ITS1 (5′-TCC​GTA​GGT​GAA​CCT​GCG​G-3′) and reverse primer ITS4 (5′-TCC​TCC​GCT​TAT​TGA​TAT​GC-3′). The polymerase chain reaction (PCR) was conducted using Phusion polymerase (Thermo Fischer Scientific, Carlsbad, CA, United States). The PCR products were then purified using the PureLink^®^ PCR Purification kit, Invitrogen (Thermo Fisher Scientific, Carlsbad, CA, United States). After purification, the amplicons for each fungal isolate were quantified using a NanoDrop 2000c (Thermo Scientific, United States) and sent to Central Analytical Facility (CAF) at Stellenbosch University (Stellenbosch, South Africa) for ITS sequencing. The quality assessment of the ITS sequences from CAF, Stellenbosch University, was performed using Snap Gene viewer (Version 6.1.2). The Basic Local Alignment Tool (BLAST) application of the nucleotide database of the National Centre for Biotechnology Information (NCBI) search program was used to generate consensus sequences from the forward and reverse ITS sequences. The consensus sequences were compared to other ITS sequences with GenBank databases through blast based on sequence similarity. The isolates PO2PL1, PO4PR1 and PO4PR2 were identified as *A. Alternaria* and deposited on the NCBI database under accession numbers OP648259, OP648254, and OP648252, respectively.

### 2.4 Extraction of endophytic fungal crude extracts

Three small fungal plugs (1 cm^2^) from 5-day-old malt extract agar (MEA, Neogen, United States) were cut. Each was inoculated into a 10 ml Potato Dextrose Broth (PDB, Neogen, United States) or Malt extract broth (Neogen, United States) in McCartney bottles and incubated in the dark at 25°C for 14 days. Each fungal crude extract was cultured in triplicates with fungal-free media as controls. After 14 days of incubation, an equal volume of absolute methanol (ChemLab supplies, South Africa) was added to each culture and placed on a rotatory shaker (Reflecta Laboratory Supplies, Glassware and Chemicals, South Africa) to shake overnight at 150 rpm at 25°C. The extracts were separated from the mycelia using gauze as a filter on pre-weighed culture flasks. The mycelia were discarded, and the supernatants were dried at 40°C. The dried methanol crude extracts were stored at −80°C until further use. Before each use, the extracts were resuspended in distilled water to a concentration of 400 μg/ml.

### 2.5 Cell cultures

TZM-bl cells derived from HeLa cells engineered to express CD4, CCR5, and CXCR4, also integrated with reporter genes from firefly Luciferase (NIH AIDS Research and Reference Reagent Programme). Cells were maintained in Dulbecco’s Modified Eagle Medium (DMEM) (Gibco, Grand Island, NY, United States) supplemented with 10% heat-inactivated fetal bovine serum (FBS) (Gibco, Grand Island, NY, United States), 50 μg/ml Gentamycin (Thermo Fisher Scientific, Carlsbad, CA, United States), and 25 mM Hepes buffer (Thermo Fisher Scientific, Carlsbad, CA, United States).

### 2.6 Generation of pseudovirus (pNL4.3) by transfection

Pseudovisuses were prepared by transfecting HEK 293T cells (10 million cells) in 10 ml growth medium (DMEM supplemented with 10% heat-inactivated fetal bovine serum, 50 μg/ml Gentamycin and 25 mM HEPES buffer) in a T75 flask with 12 μl of *env* deleted pNL4.3*luc* (donated by Dr. Katlego Sojane) and 12 μl of both backbone recombinant plasmids (pCMV and pVSV-g) using X-tremeGENE^™^ HP DNA Transfection Reagent (Sigma-Aldrich^®^ Solutions, Germany) according to the manufacturer’s protocol. The mixture of pNL4.3Δenv and pCMV, pVSV-g was incubated for 30 min at room temperature to promote DNA transfection complex formation. The DNA transfection complex was added dropwise into a 6-well plate and incubated at 5% CO_2_, 37°C, for 48 h. After that, supernatants were harvested using a syringe and 0.22 μm filter and were filtered into new tubes after 48 h for storage at −80°C freezer. The pseudoviruses’ 50% tissue culture infective doses (TCID_50_) were determined by serially diluting the virus and infecting the TZM-bl cell lines. The concentration that elicits a 50,000 relative luminescence unit was determined using the luciferase assay (Victor Nivo multimode plate reader; PerkinElmer Inc. United States).

### 2.7 Screening of the cytotoxic effect of the endophytic fungal crude extracts using MTT assay

The cytotoxicity effect of the endophytic fungal crude extracts was assessed on TZM-bl cell lines using the MTT (3-[4.5-dimethylthiazol-2-yl]-25 diphenyl tetrazolium bromide) cell proliferation assay kit according to the manufacturer’s instruction (ThermoFisher Scientific, South Africa) (Kumar et al., 2018).

Briefly, TZM-bl cells were seeded (10,000 cells/well) in a 96-well plate and incubated at 37°C, 5% CO_2_. Ten microliters of each crude extract were serially diluted 10-fold from 300 μg/ml in DMEM containing 10% heat-inactivated fetal bovine serum, 50 μg/ml Gentamycin, and 25 mM Hepes buffer in a 96-well plate. Azidothymidine (AZT) at 300 μg/ml was used as the positive control and uninfected cells as the negative control. The plates were covered with foil and incubated for 48 h at 37°C, 5%, CO_2_. After incubation, 10 µl MTT reagent (5 mg/ml in PBS) was added to each well and incubated for 4 h, 37°C, 5% CO_2_. Afterwards, the media was replaced with a fresh DMEM medium. Then, the formazan crystals were dissolved in 50 µl 0.2% DMSO and incubated for 10 min. The absorbance was measured using a Victor Nivo, multimode plate reader at 540 nm (PerkinElmer Inc. United States) (Mossman, 1983). The results were expressed as the percentage viability of cells. The cytotoxicity concentration at 50% (CC_50_) for each extract was calculated based on the non-fit regression curve on the GraphPad Prism Software (v.5.00.288). The percentage cell viability was calculated using the formula:

$$\left(\%\right)Cell\;Viability=\frac{\left(Sample\;absorbance-Cell-free\;sample\;blank\right)}{\left(Mean\;media\;control\;absorbance\right)}\times 100$$

### 2.8 Antiviral screening of endophytic fungal crude extracts

#### 2.8.1 Luciferase-based antiviral assay

The luciferase-based antiviral assay was performed according to Cherne et al., 2019 (Cherne et al., 2019). Briefly, the TZM-bl cell lines were maintained at 37°C and 5% CO_2_ in DMEM medium (containing 10% heat-inactivated fetal bovine serum, 50 μg/ml Gentamycin, and 25 mM Hepes buffer) (Sarzotti-Kelsoe et al., 2014). In this experiment, 10 µl of each crude extract was diluted 10-fold from 300 to 0.03 μg/ml in DMEM in a 96-well plate to achieve varying crude extract concentrations. After that, 50 µl HIV-1 NL4.3 virus (400 TCID) was added to all wells except cell control wells and incubated for 1 h at 37°C, 5% CO_2_. Then, TZM-bl cell suspension prepared at a density of 10,000 cells/ml in DMEM containing Dextran (Thermo Fisher, South Africa) was seeded (10,000 cells/well) in a 96-well plate and incubated at 37°C, 5% CO_2_ for 72 h. Positive control was set up using a known reverse transcriptase inhibitor, azidothymidine (AZT), at a 300 μg/ml starting concentration. Negative control was set up using uninfected cells. Cells infected with the virus without treatment were also included in the experiment (virus control). After incubation, the DMEM medium was replaced, and 100 µl BrightGlo luciferase reagent (Promega, Madison, United States) was added to each well under low light conditions and incubated at room temperature for 2 min to allow complete cell lysis. All the contents were transferred to a corresponding 96-well bottom flat black plate (Costar, Germany). Luminescence was read immediately in a Victor Nivo multimode microplate reader at 540 nm (PerkinElmer; United States). The level of viral replication was expressed as a percentage of the HIV-1 inhibition following this equation:

$$\left(\%\right)HIV\;inhibition=\frac{\left(Average\;sample-Average\;control\right)}{\left(1-\left(Average\;viral\;control-Average\;control\right)\right)}\times 100$$

The half-maximal inhibitory concentration (IC_50_) was calculated using GraphPad Prism Software (v.5.00.288).

#### 2.8.2 HIV-1 inhibition in peripheral blood mononuclear cells (PBMCs) and CD4^+^ T cells

Peripheral blood mononuclear cells (PBMCs) were isolated from the blood of HIV-1 uninfected donors at the HIV Pathogenesis Programme (HPP) sample repository. Peripheral blood mononuclear cells were isolated from 10 ml of blood using the Ficoll-Histopaque gradient method. The cells were cultured in R-10 (RPMI-1640 Gibco, Grand Island, NY, United States) supplemented with 10% heat-inactivated fetal bovine serum (FBS) plus 100 μg/ml Penicillin/Streptomycin (Gibco, Grand Island, NY, United States) and 1 M HEPES buffer.

Human CD4^+^ T cells were isolated from PBMCs using EasySep™ Human CD4^+^ T Cell Isolation Kit according to manufacturer instructions (Stemcell Technologies; Canada). A total of 10 × 10^6^ cells/ml [in interleukin-2 (IL-2) growth medium] were stimulated with 10 μg/ml phytohaemagglutinin (PHA) (Sigma-Aldrich, Millipore, Roche) in a cell culture T25 flask at 37°C, 5% CO_2_ for 3 days. After the incubation period, the cells were centrifuged at 1,000 rpm for 10 min, and the cell pellet was washed twice with PBS. The final stimulated cell pellet was resuspended in an RPMI-1640 supplemented with an IL-2 growth medium.

The CD4^+^ T cells and PBMCs were prepared in RPMI-1640 (Sigma) medium growth supplemented with penicillin/streptomycin plus 10% fetal calf serum (Wang et al., 2018). They were plated in each well (100,000 cells/well) of a 24-well culture plate, followed by the addition of 100 μl of the 100 μg/ml crude extracts. After that, 100 µl HIV-1 NL4.3 virus was added to all wells except for the negative control before the plate was incubated at 37°C, 5% CO_2_ for 48 h. Azidothymidine (AZT) at 300 μg/ml was used as a positive control, and uninfected cells were used as negative controls. After incubation**,** the plate was centrifuged at 2,400 rpm for 10 min, and 1,000 μl of culture supernatant was collected every 3 days up to day 12. Old media was replaced with fresh RPMI-1640 media (Sigma) supplemented with penicillin/streptomycin plus 10% fetal calf serum and used for determining the p24 antigen levels. The HIV-1 viral load in the culture supernatant was measured using the Quicktiter Lentiviral Quantification kit (Cell Biolabs Inc., San Diego, CA, United States) following the manufacturer’s instructions. The multimode microplate reader measured the p24 level at 480 nm (PerkinElmer Inc. United States). The viral titer was calculated as per the manufacturer’s instructions as follows:

The average genome size of lentivirus is 8 kbp, therefore.

1 ng lentiviral RNA = (1 × 10^−9^)g/(8,000 bp × 660 g/bp) X 6 × 1,023 = 1.1 × 108 VP

Virus Titer (VP/ml) = Amount of lentiviral RNA (ng) X 1.1 × 108 VP X (20 μl/5 μl)

Viral sample volume (ml)

Virus Titer (VP/ml) = Amount of lentiviral RNA (ng) X 4.4 × 108 VP/ng.

Viral sample volume (ml).

The p24 core protein concentration was calculated using GraphPad Prism (v.5.00.288).

#### 2.8.3 Solid phase extraction

Solid phase extraction (SPE) was performed following a method described by Abdah et al. (2014), Cutignano et al. (2015), and Kiełbasa et al. (2019) with some slight modifications. Endophytic fungal crude extracts were dried at 40 °C followed by sequential fractionation steps. Briefly, the removal of lipids was achieved by hexane/water extraction 50/50 (v/v), 4-ml total volume, and an aqueous phase was collected and dried. After drying, the crude extracts were reconstituted in 50% HPLC grade methanol (MeOH, Sigma Aldrich, South Africa), and 1 ml of this solution was spiked with 20 µl of phosphoric acid (Sigma Aldrich, South Africa) and loaded onto HLB (Hydrophilic-Lipophilic Balance), MCX (Mixed-mode, strong Cation-eXchange), and MAX (Mixed mode, strong Anion-eXchange) cartridges obtained from Waters Corporation (Prague, Czech Republic). The adsorbed compounds were desalted and stepwise eluted with increasing (5%, 45%, and 95%) organic solvent (MeOH), providing sample variants, respectively. Eluted fractions of organic compounds were concentrated by evaporation to dryness at 40°C under a gentle stream of nitrogen. Dried fractions were reconstituted with 1 ml of acetonitrile (Sigma Aldrich, South Africa) and filtered with a 0.2 µm filter. HLB was based on N-vinylpyrrolidone–divinylbenzene copolymer. MCX was a cation-exchange sorbent representing the HLB material modified with SO3H− groups. MAX was an anion-exchange cartridge. After the first round of fractionation, all samples were tested for anti-HIV-1 activity using a luciferase-based antiviral assay as described above. The fractions were sent to the University of KwaZulu Natal Pietermaritzburg Campus, School of Chemistry and Physics for Gas Chromatography-Mass spectrometer characterization.

#### 2.8.4 Gas chromatography-mass spectrometer (GC-MS)

Analysis of fractions was carried out using a Hewlett Packard 6890 (United States) GC-MS fitted with a Zebron ZB-5MSplus column (30 m × 025 mm × 0,2.5 μm) following a modified protocol based on Mishra and Patnaik, (2020). Oven temperature was set at an initial temperature of 35°C with a hold time of 3 min. The temperature was programmed to ramp up by 8°C/min to 280°C followed by 10°C/min to a 5 min hold. The fractions were injected at 1 μl at a splitless injection (spilt 20:80-8-200-5M-8-260-10M10-280-HP5-ETOH) and a flow rate of 1 ml/min with Helium as a carrier gas and the spetra was captured at 70 eV. Following the separation in the column, the components were identified and evaluated using a Flame Ionization Detector (FID). Compounds were identified by comparing the spectrum of unknown compounds to the spectrum of known compounds in the NIST MS 2.0 structural library to determine their names, molecular weight, and structure 53.

### 2.10 Statistical analysis

The results of all the experiments were the average of three independent experiments. Analyses of concentration-response data were performed with a non-linear curve-fitting program in GraphPad Prism to determine CC_50_ and IC_50_ values.

## 3 Results

### 3.1 Molecular identification of the endophytic fungi isolates

**FIGURE 1:**
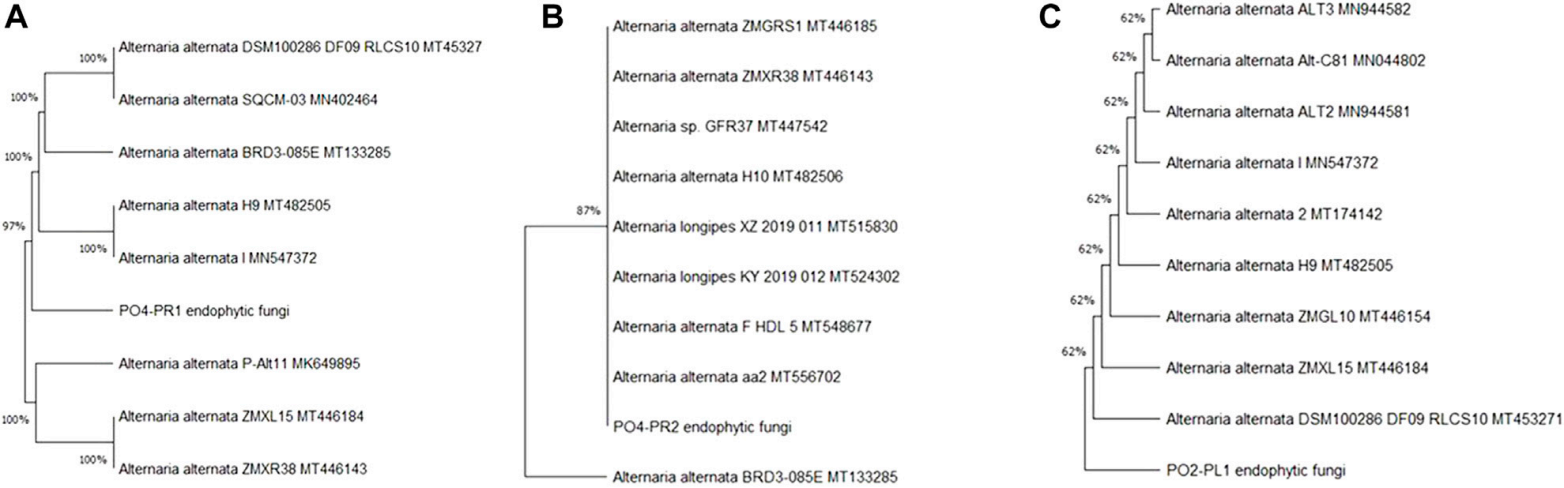
Phylogenetic tree of isolated endophytic fungi from *S. birrea*, and *Hypoxis* species, **(A)** PO4-PR1. **(B)** PO4PR2 **(C)** PO2PL1. Taxonomic classification was represented by the maximum-likelihood tree relationships of aidophytic fungal isolates to other sequences from NCB]. Bootstrap values for (1,000 replications) are displayed on the branches of the trees.

In this study we isolated fifteen endophytic fungi from two plants and only three of these isolates exhibited anti-HIV activities and these were identified through internal transcribed spacer (ITS) sequencing with 350–400 bp. The fungal ITS sequences (PO4PR1, PO4PR2 and PO2PL1) were trimmed and analyzed using the SnapGene viewer, version 6.1.2 (GSL Biotech LLC, Chicago, IL, United States). The forward and reverse sequences were aligned to produce consensus sequences and submitted to National Center for Biotechnology Information (NCBI) Basic Local Alignment Search Tool (BLASTN) for identifying similar sequences (Treves, 2010). The results from NCBI identified all three fungal isolates (PO2PL1, PO4PR1, and PO4PR2) as *A. alternata* (Table 1). Phylogenetic trees for the best hits were constructed in MEGA X version 11 (Kumar et al., 2018). Per the phylogenetic trees and BLAST ID, endophytic fungi POPR1 was closely related to *A. alternata* by 99.81% (Figure 1A). PO4PR2 strain was closely related to *A. alternata* by 99.80% (Figure 1B), and PO2PL1 was assigned as *A. alternata* with a close relationship at 99.81% (Figure 1C).

**TABLE 1:** Endophytic fungi isolated from *S. birrea* and *Hypoxis* species.

Lab code	Blast ID	Percentage identity (%)	Query coverage (%)
PO4-PR1	*A. alternata*	99.81	100
PO4-PR2	*A. alternata*	99.80	100
PO2-PL1	*A. alternata*	99.81	100

### 3.2 The cytotoxicity effects of endophytic fungi crude extract on TZM-bl cell lines

**FIGURE 2:**
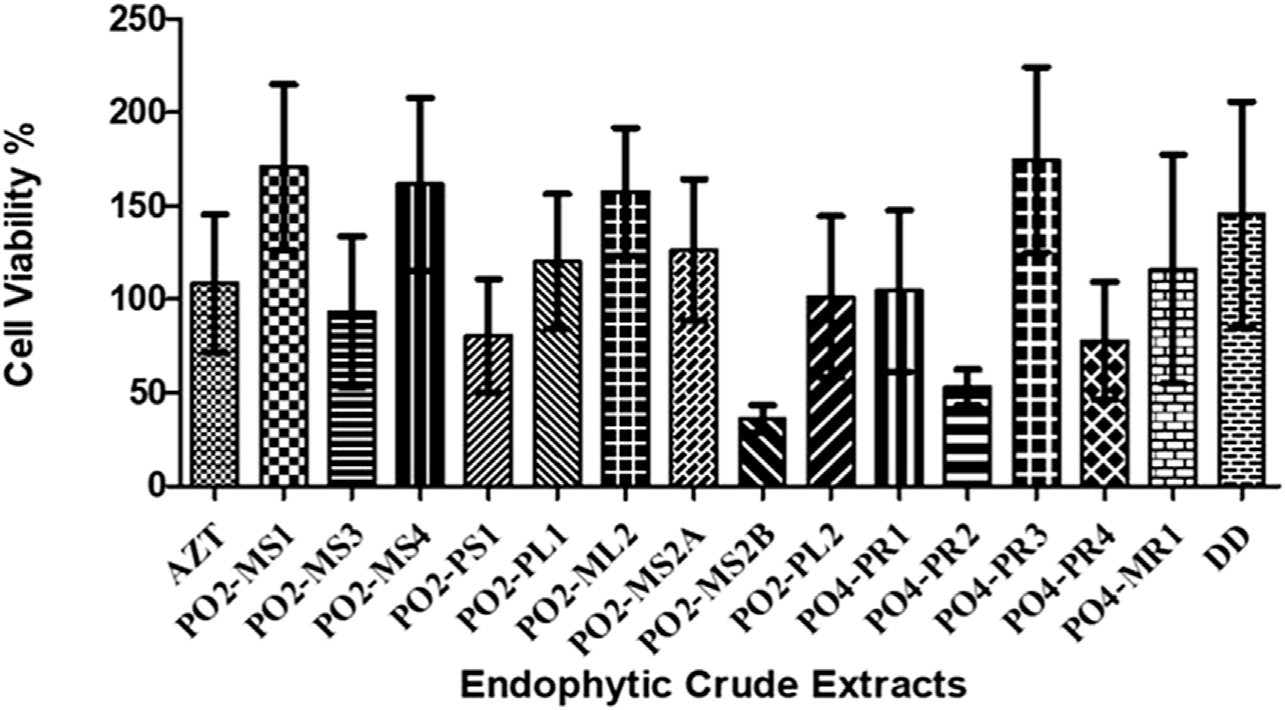
Percentage of cell viability of TZM-bI cell lines against eudophytic fungi crude extracts. The endophytic fungi was isolated from the roots, bark and stem of the *S. birrea*, and *Hypoxis* species. All the crude extract were not toxic to the cell. Results were obtained from three independent experiments: data are mean SEM.

The cytotoxicity analysis was performed on TZM-bl cells using MTT proliferation to test the cytotoxicity effects of the crude extracts of all 15 fungal crude extracts on TZM-bl cells. The cytotoxicity of each crude extract was represented as the percentage of cell viability (Figure 2). The *A. alternata* PO2MS2B strain had a cell viability of less than 50%, followed by *A. alternata* (PO4PR2), which was above 50%. According to the United States National Cancer Institute Plant Screening Program, a crude extract is generally considered to have *in vitro* cytotoxic activity when the CC_50_ value is < 30–40 μg/ml (Talib and Mahasneh, 2010). Only two strains, *A. alternata* PO2MS1 and PO2MS4, met this requirement for TZM-bl cell lines. The cytotoxicity results indicate that the remaining 13 endophytic crude extracts showed no cytotoxicity on TZM-bl cells with CC_50_ values ranging from 41.25 to 94 μg/ml (Table 2). The positive reference, AZT, did not show cytotoxicity on TZM-bl cells at a CC_50_ value of 35.64 μg/ml. The CC_50_ is significant in these results to exclude the non-specific antiviral effect and decide the extracts’ toxic concentration.

**TABLE 2:** Cytotoxic concentrations (CC_50_) of all extracts on TZM-bl cells.

Extract	CC_50_ (μg/ml)
AZT (Control)	36.64
PO2-MS1	35.74
PO2-MS3	45.44
PO2-MS4	38.43
PO2-PS1	42.13
PO2-PL1	45.74
PO2-ML2	45.83
PO2-MS2A	41.25
PO2-MS2B	46.64
PO2-PL2	85.23
PO4-PR1	45.42
PO4-PR2	43.5
PO4-PR3	47.13
PO4-PR4	49.44
PO4-MR1	48.58
DD (PO2ML3)	93.30

### 3.3 The anti-HIV-1 effects of the endophytic fungi crude extract on using a luciferase-based antiviral assay

**FIGURE 3:**
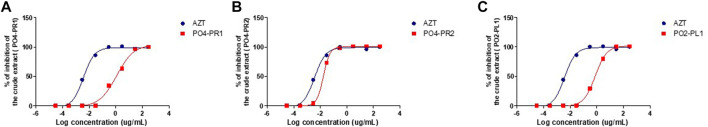
Percentage inhibition curves of the endophytic fungi crude extracts tested with Luciferase-based antiviral assay using TZM-bl cell lines. The TZM-bl cells were infected with HIV-1 (NL4.3) and treated with the serial dilution of endophytic fungi crude extracts with AZT as positive control. The TZM-bl cells (treated and untreated) were incubated for 48 h at 37°C and 5% CO_2_. **(A)** *A. alternata* PO4PR1 isolated from roots of *Hypoxis* species (ICso = 1.170 μg/mL) **(B)** *A. alternata* PO4-PR2 isolated from roots of *Hypoxis* species (ICso = 0.017 μg/mL), and **(C)** *A. alternata* P02-PL 1 isolated from leaf of *S birrea* (IC50 = 0.766 μg/mL).

The luciferase-based antiviral assay was performed to determine the effect of the endophytic fungi crude extracts on the TZM-bl cells, and the results were normalized against the untreated cells. Three crude extracts of *A. alternata* (PO4PR1, PO4PR2, and PO2PL1) out of 15 screened endophytic fungal crude extracts showed antiviral activity against HIV (Figure 3). The remaining 13 crude extracts had no significant inhibitory effect on TZM-bl cells as they did not reach at least 50% inhibition at the varying ten-fold dilutions (results not shown). The positive control, AZT, showed 50% inhibition at a concentration of 0.004 μg/ml. The crude extract of *A. alternata* PO4PR1 showed 50% inhibition HIV-1 replication in TZM-bl cells at 1.170 μg/ml (Figure 3A)*, A. Alternata* PO4PR2 showed 50% inhibition at 0.017 μg/ml (Figure 3B) and *A. alternata* PO2PL1 at 0.766 μg/ml (Figure 3C). The IC_50_ values for each crude extract are given in Table 3. Furthermore, the selective index (SI) was calculated for all three crude extracts to establish the most potent activity. The selective index for PO4PR1, PO4PR2, and PO2PL1 were 38.82, 2558.82, and 59.71 μg/ml, respectively.

**TABLE 3:** Inhibition concentration (IC_50_) and cell cytotoxicity of *A. alternata* extracts in Luciferase based assay. The selective index was determined by dividing the cell cytoxicity and inhibitory concentration at 50%.

Extract	IC_50_ (µg/ml)	CC_50_ (µg/ml)	SI
AZT (control)	0.004	36.64	9,160
PO4-PR1	1.170	45.42	38.84
PO4-PR2	0.017	43.5	2,558.82
PO2-PL1	0.766	45.74	59.712

### 3.4 HIV-1 virus associated p24 antigen assay

**FIGURE 4:**
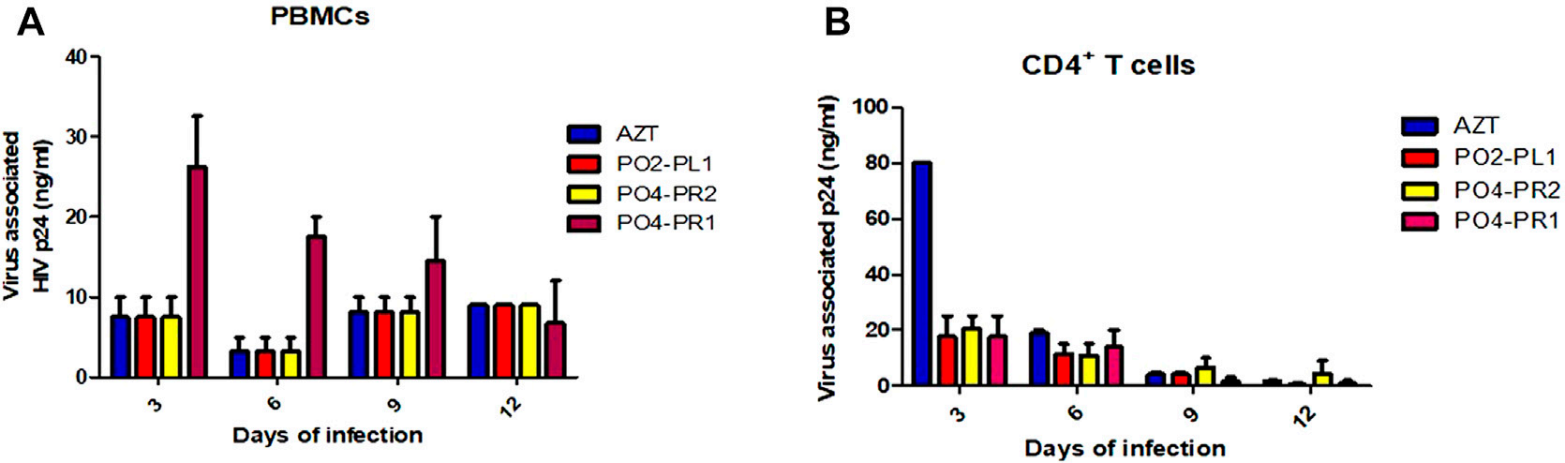
Anti-HIV activity of crude extracts of *A. alternata* PO4PR1, PO4PR2, and PO2PL1 as determined by p24 enzyme-linked immunosorbent assay (ELISA). *A. alternata* PO4-PR1 and PO4PR2 were isolated from *Hypoxis* spp. root and *A. alternata* PO2PL1 isolated from *S. birrea*’s leaves. The effect of the crude extracts (100 μg/mL) on HIV-1 replication was determined by infecting **(A)** PBMCs with HIV-1 NL4.3 in the presence of test crude extracts and **(B)** CD4^+^ T cells with HIV-1 NL4.3 in the presence of test crude extracts. AZT (positive control) was included at 100 μg/mL, *A. alternata* PO4PR1 (1.170 μg/mL), *A. alternata* PO4PR2 (0.017 μg/mL) and *A. alternata* PO2PL1 (0.776 μg/mL). The p24 antigen levels in all culture supernatants were determined and expressed as the concentration of HIV-1 p24 expression in every single experiment.

All three crude extracts inhibited virus replication at concentrations ranging from 0.3 to 50.2 μg/ml in PBMCs. The crude extract of *A. alternata* PO4PR2 had the highest p24 expression of 50.2 ng/ml after infection, followed by *A. alternata* P02PL1 at 24.6 ng/ml and *A. alternata* P04PR1 at 22.1 μg/ml in PBMCs (Figure 4A). In all three tested crude extracts, the commercial reverse-transcriptase inhibitor AZT was tested as a reference drug and expressed p24 antigen at 50.7 μg/ml, which is highly similar to that of *A. alternata* PO4PR2 in PBMCs. The levels of p24 expression started decreasing on day six in all supernatant cultures. In the case of crude extracts on CD4^+^ T cells, the levels of p24 antigen in the virus culture supernatants ranged from 0.1 to 34.6 μg/ml upon infection (Figure 4B). The crude extracts of *A. alternata* PO4PR2 and *A. alternata* PO2PL1 showed HIV-1 inhibition as the p24 levels were deficient or not expressed in culture supernatants from days nine to twelve.

### 3.5 Gas chromatography-mass spectrometer (GC-MS)

**FIGURE 5:**
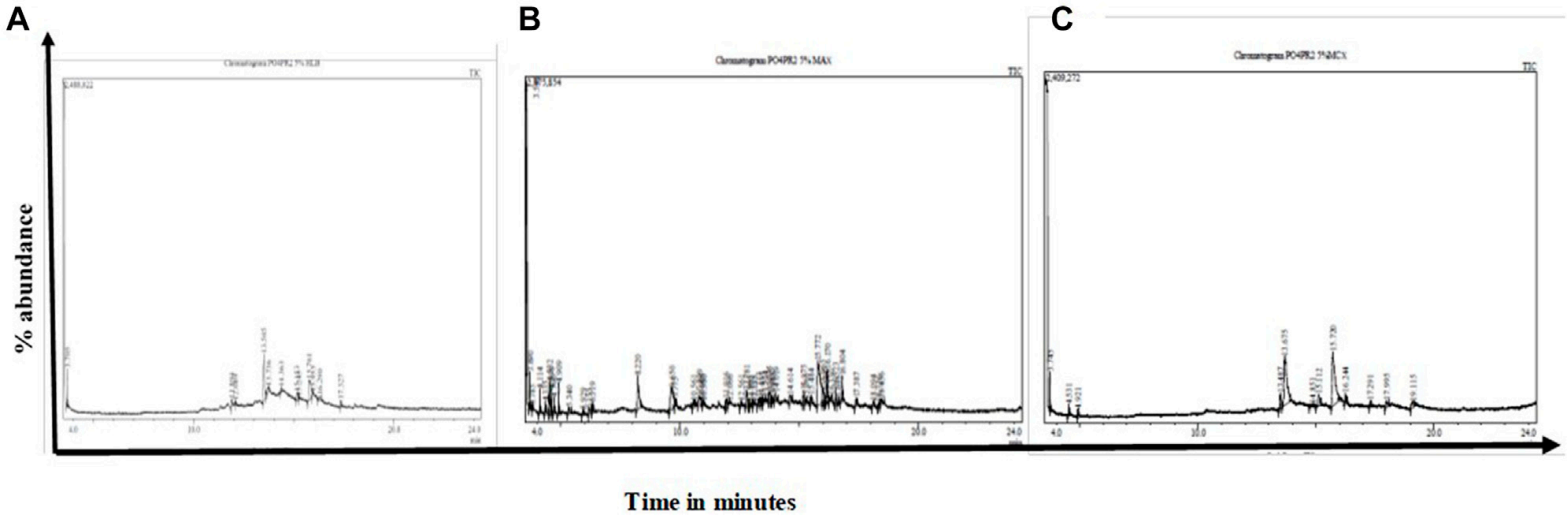
GC-MS chromatographs of the endophytic fungi fractions from *A. alternata* (PO4PR2) **(A–C)** showed indicate chromatographs of the **(A)** HLB cartridges fractions; **(B)** MCX cartridge **(C)**, MAX. The fractions were eluted from the different cartridges using 5% methanol. PO4PR2 was isolated from *Hypoxis* species root and has high selective index.

The GC-MS analysis of fractions generated revealed 48 compounds (Figure 5). Table 4 summarizes fifteen compounds with a more than 80% similarity index. The most prevalent compounds in all the extracts were cyclotrisiloxane octamethyl (22.92%); Propaninitrile (16,67%); Pyrrolo[1,2-a]pyrazine-1,4-dione, hexahydro-3-(2-methyl propyl) (10.42%); Silane, diethylethoxy(2-ethoxyethyloxy) (4. 17%); and 1,2-Cyclobutanedicarbonitrile (2.08%) with reported biological activities such as antimicrobial, anti-inflammatory and antioxidant properties.

**TABLE 4:** GC-MS analysis of secondary metabolites of endophytic fungi PO4PR2 (*A. alternata* isolated from *Hypoxis* plant crude methanol extracts) separated using HLB, MCX and MAX cartridges.

Similarity index (%	Retention time (min)	Name of compound	Biological activity/properties	Natural product sources	References
95	3.660	Propanenitrile	Antimicrobial	*Brassica rapa*	Makawana et al. (2011)
					LOTUS- the natural products occurrence data
87	3.540	1,2-Cyclobutanedicarbonitrile	Anti-inflammatory; anti-depressant, liquid crystal properties; anticancer; antiviral	*Agelas sceptrum* (sceptines) of the sea sponge	Demir et al. (2012)
					Van der Kolk et al. (2022)
94	6.300	Cyclotetrasiloxane, octamethyl-	Anti-oxidants; Anti-microbial; antifungal	Extract of the olive leaves (*Olea europaea. L.*)	Ismail et al., (2019); Jayakumar et al. (2021)
				*Pulicaria undulata L)* extrac*t*	Keskin et al. (2012)
					Helal et al. (2019)
92	3.625	Proparglylamine	Transcription repressing activity	NR	Maiques-Diaz and Somervaille, (2016)
85	8.192	Cyclopentasiloxane, decamethyl-	Antimicrobial; aromatherapy; Pharmacological	Extract of the olive leaves (*Olea europaea. L.*)	Keskin et al. (2012)
89	3.625	2-butyne	Antimicrobial	Not reported	Tatsumi et al. (2001)
89	6.573	2,5-Dihydrothiophene sulfone	Pharmacology	Not reported	Mustafa et al. (2020)
88	3.660	Cyclobutanecarbonitrile	Aroma; Human whole blood IDO1 inhibition activity	*Agelas sceptrum* (sceptines) of the sea sponge	Yu et al. (2021)
					Van der Kolk et al. (2022)
88	3.660	Butanetrile, 4-oxo	Anti-depressants	Not reported	Zhang et al. (2021)
85	6.300	1,3-Cyclobutanedicarbonitrile	Anti-inflammatory; anti-depressants; anti-parasitic	*Agelas sceptrum*	Koparir et al. (2022)
					Van der Kolk et al. (2022)
85	3.540	1,2-butadiene	Anti-bacterial; anti-fungal	Not reported	Avery et al. (2007); Zheng et al. (2015)
82	6.300	2,6-Dihydroxyacetophenone, bis(trimethylsilyl) ether	Anticancer	*Parthenium hysterophorus*	Pradhan and Dubey, (2021)
			Antimicrobial activity; antifungal		Krishnaveni et al. (2015)
81	8.180	Benzeneethanamine	Anti-inflammatory; antimicrobial	*Chryseobacterium polytrichastri*	Chinaka et al. (2018)
					LOTUS- natural product occurrence database
85	3.500	1-butyne	anti-bacterial; anti-fungal; genotoxic activity	Not reported	Kitamura et al. (2002)
88	18.089	Pyrrolo[1,2-a]pyrazine-1,4-dione, hexahydro-3-(2-methyl propyl)-	Aflatoxin production inhibition	*Streptomyces xiamenensis, Vibrio anguillarum, and Ophiocordyceps sinensis*	Sahadevan et al. (2014)
					LOTUS- natural product occurrence database
61	16.79	Coumarin, 3,4-dihydro-4,5,7-trimethyl-4,5,7-Trimethyl-2-chromanone	Antiviral activity	*Tabebuia Argentea*	Govindappa et al. (2015)
			HIV-1 integrase, HIV-1 reverse transcription	*Calophyllum inophyllum*	Govindappa et al. (2015)

## 4 Discussion

There is a concerted effort to search for new antiretroviral drugs to inhibit or eliminate HIV and suppress it from reactivation (Mukhtar et al., 2008; Ghildiyal et al., 2020). This study investigated the anti-HIV-1 activity of endophytic fungi isolated from *S. birrea* and *Hypoxis* plants. Three of the fifteen endophytic crude extracts demonstrated anti-HIV-1 activities when screened on TZM-bl cell lines, PBMCs, and CD4^+^ T cells from uninfected HIV donors suggesting that endophytic crude extracts isolated from *S. birrea* and *Hypoxis* plants may be potential sources of novel bioactive compounds with possible anti-HIV activity.

The three bioactive fungal isolates were all identified as *A. alternata* using Blastn search of the ITS1/4 rDNA sequence. The ITS region is widely accepted in the mycology community as the DNA barcoding marker with the highest probability for the precise identification of a broad range of fungal species (Schoch et al., 2012; Raja et al., 2017). *A. alternata* species are commonly isolated in plants existing as endophytes or pathogens and they display varied symbiotic lifestyles and wide host ranges (Lou et al., 2013; De Mers, 2022). Fungal species such as *A. alternata* which are broadly delineated due to their unstable taxonomy and limited morphological characteristics for species delimination, are often further delineated using phylogenetic analysis. In our phylogenetic analysis of the three fungal isolates it was revealed that these isolates closely cluster with *Alternaria* species. However, for *A. alternata* there are few clades, thus limiting the power of further species delineation using evolutionary relationship (De Mers, 2022). Endophytic isolates of *A. alternata* have been shown to exist as asymptomatic symbionts with no apparent ability to cause plant disease (Spurr and Welty, 1975). While *Alternaria* species has been reported as an endophyte in other plant species, this is the first study to report *A. alternata* endophytes from *S. birrea* and *Hypoxis* plants. *Alternaria* species in endophytic lifestyle have been shown to exhibit anti-HIV activities and some of the active compounds and their mechanism of action have been elucidated (Wellensiek et al., 2013; Eram et al., 2018). For example, in a study by Bashyal et al. (2014) they isolated alterotoxins from *A. tenuissima* and Govindappa et al. (2015) isolated coumarins from *Alternaria* species.

In the current study, we first determined the cytotoxic concentration of the isolated *A. alternata* species against TZM-bl cell line starting with a concentration of 300 μg/ml and diluted 10 fold to a concentration of 0.03 μg/ml. Most of our investigated crude extracts showed no cytotoxicity on TZM-bl cells, with CC_50_ values ranging from 41.25 to 94 μg/ml. The positive reference, AZT, did not show cytotoxicity on TZM-bl cells at a CC_50_ value of 35.64 μg/ml. A crude extract with a CC_50_ value < 30–40 μg/ml is generally considered to have *in vitro* cytotoxic activity, while those with CC_50_ > 40 μg/ml are considered not to have a cytotoxic effect (Talib and Mahasneh, 2010). A recent study has shown that crude extracts of *A. alternata* did not possess cytotoxic activity against the Vero cell line as the CC_50_ was greater than 4,000 μg/ml (Elghaffar et al., 2022). Therefore, this suggests that at this maximum tested concentration (300 μg/ml), *A. alternata* crude extracts remain safe for use on TZM-bl cell line models.

In our study, crude extracts of *A. alternata* were assessed for HIV inhibition using the Luciferase based assay. Results revealed that crude extracts of *A. alternata* exhibited promising HIV-inhibition with inhibitory concentrations (IC_50_) ranging from 0.017 to 1.170 μg/ml. Bashyal et al. (2014) reported an HIV-1 inhibition value of 0.9 µM from alterotoxin V of *Alternaria tenuissima*. Ma et al. (2013) reported anti-HIV reverse transcriptase activity of a PHP protein isolated from *Peganum harmala* seeds with 50% inhibitory concentration at 1.5 µM. Govindappa et al. (2015) revealed that partially purified coumarins isolated from fungal endophytes, *Alternaria* sp. displayed high inhibition on three viral enzymes, HIV-1 Reverse Transcriptase, which was inhibited by 82.81% (control, heparin = 74.54% inhibition), integrase enzyme activity which was inhibited by 98% and protease activity was inhibited by 78%. Most studies have tested *A. alternata* against bacterial strains for activity. Hence there are not many published reports on anti-HIV activity. Qader et al. (2021) reported that crude extracts of *A. alternata* had antimicrobial effects against all tested bacteria with minimum inhibitory concentrations between 2.5 and 5 μg/ml, slightly higher than our observed activity. From CC_50_ and IC_50,_ the selective index of the three isolates, PO4PR1, PO4PR2, and PO2PL1, were determined to be 38.82, 2558.82, and 59.71, respectively. Among all three endophytic extracts, PO4PR2 showed the most significant virus inhibition as it had the highest selective index. These results demonstrated that crude extracts of *A. altenata* have a broad spectrum of biological activities and provide an opportunity for further anti-HIV drug development from natural products.

To further confirm the HIV-inhibition activity, crude extracts of *A. alternata* in all three isolates showed effective antiviral activity compared to the standard drug AZT in HIV p24 antigen inhibition assay. *A. alternata* PO4PR1 showed the lowest p24 concentration at 22.1 ng/ml, revealing that there is strong anti-HIV activity. On the other hand, *A. alternata* PO4PR2 showed a higher p24 concentration at 50.2 ng/ml in PBMCs, meaning that the HIV-inhibition activity is low. The PO2PL1 extract showed inhibition at 24.6 ng/ml after 3 days of infection. Methanol extracts of all tested fungi, *A. alternata* showed a weak HIV-1 inhibition of less than 50 ng/ml in CD4^+^ T cells after infection. The inhibition rate was less than 50 ng/ml after infection. All the methanol extracts revealed a gradual decrease of p24 antigen expression in each, indicating potential denaturation of virus (virucidal activity) or HIV-1 inhibitory activity. Perhaps this decrease might also be caused by the instability in the cell culture medium, which was replaced after every collection of the supernatant. Similar results were reported by Subramaniam et al. (2020) using a different endophytic fungus on PBMCs. Methanolic extracts (100 μg/ml) of *Padina tetrastromatica* inhibited HIV-1 by more than 50% and showed a significant reduction in HIV-1 p24 levels after 7 days of incubation. The methanol extract of *Alternaria* species inhibited the activity of HIV-1 Reverse Transcriptase (82.81 ± 1.0) (Govindappa et al., 2015). The low level of p24 in the cell culture of PBMC and CD4^+^ T cells indicates the antiviral activity elicited by the *Alternaria* crude extract, which confirms the results obtained from the luciferase-based antiviral activity (Figure 3).

The GC-MS analysis of *A. alternata* crude extracts identified different compounds which might be responsible for the anti-HIV activities. The major bioactive compounds with a similarity index of more than 80% are listed in Table 4. The aliphatic nitrile and propane nitrile (16.67%) has been reported as antimicrobial agents (Makawana et al., 2011). In addition, the compound propanenitrile was found to be active against Gram-positive bacteria *Bacillus subtilis* and *Clostridium tetani* (Makawana et al., 2011). The cyclic, unsaturated Cyclopentasiloxane, octamethyl (22.92%) has been previously reported as an antimicrobial agent (Rizwana et al., 2016), antioxidant activity (Ismail et al., 2019) and plays a role in scavenging free radicals (Prakash and Suneetha, 2014). The 1.2-cyclobutanedicarbonitrile (2.08%) has been known for its anti-inflammatory, anti-depressant, and liquid crystal properties (Li et al., 2020). Proparglylamine has been known to play a role in repressing transcription activity (Marques-Diaz and Somerville 2016). Benzeneethanamine (2.23%), 2-butyne(1.80%), Cyclopentasiloxane, and decamethyl- (14.59%) were previously reported for their antimicrobial activity (Keskın et al., 2012). Pyrrolo[1,2-a]pyrazine-1,4-dione, hexahydro-3-(2-methyl propyl)-(10.42%) have been reported to have antioxidant properties and antimicrobial activities, thus have a potential future in agriculture (Sahadevan et al., 2014). Most of the compounds identified in this study have antimicrobial activity, which shows the possibility that the crude extracts portray anti-HIV activities. To our knowledge, this is the first study to report for the first time endophytic fungi associated with *A. alternata*, with anti-HIV activity against TZM-bl cell lines and primary cell lines, PBMCs, and CD4^+^ T cells. Finally, the identity of the positive hit compound from the identified volatile secondary metabolites needs to be further characterized. This study provided early hit stages and has the potential for development into hit-lead stages with more experiments on compound characterization and *in vivo* studies.

## 5 Conclusion

In the current study, our results showed that *A. alternata* isolated from *S. birrea* and *Hypoxis* harbour diverse compounds with promising anti-HIV activities and no toxicity against different human cell lines. The anti-HIV activities of the crude extracts show the potential use of endophytic fungal crude extracts. Thus, they should be considered novel sources to isolate and produce pure bioactive compounds. Future studies should be conducted to purify and chemically characterize the active compound(s) and elucidate a mode of action to establish a precise HIV-1 inhibition mechanism of the bioactive compound(s) from *A. alternata* crude extracts in this study.

## Data Availability

The datasets presented in this study can be found in online repositories. The names of the repository/repositories and accession number(s) can be found below: https://www.ncbi.nlm.nih.gov/, OP648259; https://www.ncbi.nlm.nih.gov/, OP648254; https://www.ncbi.nlm.nih.gov/, OP648252.
